# Aerobic Exercise as an Adjunct Therapy for Improving Cognitive Function in Heart Failure

**DOI:** 10.1155/2014/157508

**Published:** 2014-07-03

**Authors:** Rebecca A. Gary, Kathryn Brunn

**Affiliations:** Nell Hodgson Woodruff School of Nursing, Emory University, Atlanta, GA 30322, USA

## Abstract

Persons with heart failure (HF) are typically older and are at a much higher risk for developing cognitive impairment (CI) than persons without HF. Increasingly, CI is recognized as a significant, independent predictor of worse clinical outcomes, more frequent hospital readmissions, and higher mortality rates in persons with HF. CI can have devastating effects on ability to carry out HF effective self-care behaviors. If CI occurs, however, there are currently no evidence based guidelines on how to manage or improve cognitive function in this population. Improvement in cognition has been reported following some therapies in HF and is thought to be the consequence of enhanced cerebral perfusion and oxygenation, suggesting that CI may be amenable to intervention. Because there is substantial neuronal loss with dementia and no effective restorative therapies, interventions that slow, reverse, or prevent cognitive decline are essential. Aerobic exercise is documented to increase cerebral perfusion and oxygenation by promoting neuroplasticity and neurogenesis and, in turn, cognitive functioning. Few studies have examined exercise as a potential adjunct therapy for attenuating or alleviating cognitive decline in HF. In this review, the potential benefit of aerobic exercise on cognitive functioning in HF is presented along with future research directions.

## 1. Introduction

Heart failure (HF) is increasing at a more rapid pace than in other cardiovascular diseases in the United States (US), largely due to an aging population and advances in the treatment of coronary artery disease [1, 2]. The highest prevalence of HF is among older adults and is estimated to be 10% among those of 75 years of age, increasing to 20% among those of over the age of 80, and this number is projected to dramatically rise over the next several decades [3, 4]. Increasingly, cognitive impairment (CI) is recognized as a prevalent and significant independent predictor of poor clinical outcomes, repeat hospitalizations, and higher mortality rates in HF [5–10]. CI inhibits the person's ability to perform effective self-care, behaviors that are essential for optimal HF management and improved quality of life. Self-care in HF is complex and encompasses prevention measures (e.g., salt and fluid restrictions, weighing daily, and medication adherence), management of symptoms (e.g., resting when fatigued), and making decisions about seeking care (e.g., seeking help when symptom severity worsens) [11]. Despite CI being an independent predictor of poor outcomes and higher mortality in HF, little is known about interventions that may attenuate cognitive decline in this population.

Exercise is well established to improve the peripheral alterations associated with HF, but little research has explored the use of exercise as an adjunct therapy for CI. Exercise is documented to increase cerebral perfusion and oxygenation by promoting neuroplasticity and neurogenesis and, in turn, cognitive functioning [12]. Neuroplasticity is the ability of the nervous system to adapt to changes in the external environment in order to maintain, recover, and optimize functioning [13]. Only one small study has examined the effect of aerobic exercise on cognitive function in persons with HF [14]. A much larger literature supporting the use of exercise as an adjunct therapy for improving CI is derived from older adults and animal models [15–21]. Because there is substantial neuronal loss with dementia and no effective restorative therapies, interventions that slow, reverse, or prevent cognitive decline are essential. This review will examine CI in HF and evidence to support aerobic exercise as a potential adjunct therapy to improve cognitive function.

## 2. Cognitive Impairment in HF

Heart failure negatively affects cognitive functioning in most domains including memory, attention, learning ability, recall, psychomotor speed, and executive functioning (see [5, 6, 9, 10]). The most common domains negatively affected by HF and aging are memory and executive function, both of which can have devastating effects on ability to carry out HF self-care behaviors and substantially lowers quality of life [9].

A large number of studies in HF have relied on global screening questionnaires such as the Mini Mental Status Examination (MMSE) to assess cognitive functioning. Global cognitive measures, however, are known to lack sensitivity for detecting subtle cognitive deficits such as mild cognitive impairment (MCI) [9]. Fewer studies in HF have measured cognitive function using objective, standardized neuropsychological batteries. The most common cognitive deficit, mild cognitive impairment (MCI), is a subtle but measurable deficit in one or multiple cognitive domains, most often memory. These deficits are greater than expected with normal aging, but lack other symptoms of dementia, such as impaired judgment or reasoning and difficulties performing activities of daily living. Mild cognitive impairment makes performing instrumental activities of daily living (IADLs) more difficult than usual (e.g., scheduling appointments, remembering medications, and managing bills) but typically does not interfere with the ability to perform many basic or instrumental activities of daily living independently. A MCI diagnosis requires abnormalities to be present on neuropsychological testing (1.5 SD below age-standardized mean) in at least one cognitive domain using objective neuropsychological testing (see [2, 7, 10]). In several observational cohort studies, persons with HF had lower performance on cognitive tests than individuals without HF [22, 23] and were twice as likely to progress to dementia compared to those without HF. Dementia, a progressive syndrome that impedes the ability to perform ADLs or IADLs, social activities, or occupational responsibilities, occurs in approximately 25% of persons with HF [24].

## 3. Etiology of CI in HF

The etiology of CI in HF has not been fully elucidated, but multiple underlying mechanisms are thought to be contributing factors. These include reduced cerebral perfusion and oxygenation, structural changes in the brain (i.e., hippocampal damage, atrophy, and loss of gray matter), and microemboli [2, 23, 25–30]. The hippocampus, amygdala, frontal lobes, and cerebellum have high plasticity and are especially vulnerable to oxygen deprivation [9]. The ability to acquire new information and generate memory is key functions of the hippocampal areas. When damaged the hippocampus impairs ability to perform essential self-care activities and routine daily tasks. Studies have shown that worse HF severity is associated with poorer cognitive function and the brain changes are reflective of ischemic injury. For instance, cerebral hypoperfusion has been shown to predict cognitive decline and the transition from MCI to more severe dementia [31]. In addition, lower scores on the MMSE were reported among those who had a left ventricular ejection fraction (LVEF) less than 30% [30], indicative of systolic dysfunction. This finding is consistent with others who show that lower LVEF, higher New York Heart Association (NYHA) class, and greater duration of HF often parallel the level of cognitive impairment observed in persons with HF [5, 6, 10].

## 4. Exercise, Cognitive Performance, and HF

Over 130 smaller studies, as well as the recent large, multisite Heart Failure: A Controlled Trial Investigating Outcomes of Exercise Training (HF-Action) trial [32], have established the safety and efficacy of moderate intensity aerobic exercise in stable HF. The effect of exercise on cognitive outcomes in HF, however, remains largely unknown [2, 18, 33–35]. One small study examined the effect of exercise on cognitive functioning and cerebral perfusion in HF [14]. Findings revealed significant improvements in 2 cognitive domains (attention and psychomotor speed), but no change was reported in cerebral perfusion; the low dose exercise regimen and lack of a true comparison group limit study interpretation. A more recent trial of stroke survivors, however, found that measures of cerebral perfusion significantly increased following moderate intensity exercise and was highly correlated with improvement in peak oxygen consumption, but cognitive function was not evaluated [36]. Evidence from other populations, including those with disabilities and chronic illness (e.g., renal failure, Parkinson's Disease, multiple sclerosis, stroke, and Alzheimer's disease), suggests that exercise improves cognition via several biological processes: its impact on increased cerebral blood flow and oxygen delivery; its influence on BDNF (thought to enhance neurogenesis); and its effect on certain neurotransmitters (see [18, 20, 21, 35, 37]). Exercise is reported to improve cerebral oxygenation following moderate intensity aerobic exercise, but few studies have examined clinical populations, including HF, or the impact on cognitive functioning [38].

## 5. Aerobic Exercise in Older Adults

Aging is often characterized with loss of both white and grey matter tissue in the prefrontal, temporal, and parietal areas of the brain and these losses are manifested as decline in cognitive functioning [39]. Most evidence supports that aerobic exercise enhances cognitive performance in healthy older adults including improvements in attention and processing speed, executive function, and memory [40]. In addition, studies also support that persons with MCI may experience greater benefit from aerobic exercise in cognitive performance especially memory function than persons who have normal cognitive function [41]. Colcombe and Kramer [42] evaluated the effects of aerobic exercise on cognitive function; this meta-analysis of 18 randomized trials demonstrated that aerobic exercise improved cognitive function in a number of domains including spatial and executive functioning. The most noted improvement, however, was in executive function indicating that aerobic exercise may have selected specificity in certain areas of the brain. The benefits of aerobic exercise training on cognitive function have also been demonstrated in older adults with dementia. In a meta-analysis by Heyn et al. [41], aerobic exercise was shown to reverse cognitive impairments in demented individuals with an overall moderate effect size of 0.57 between the exercise and control groups. Another study reported that individuals in the early stages of Alzheimer's who were more physically fit had less brain atrophy compared to those less physically fit [43]. These findings suggest that aerobic exercise is beneficial for persons with and without cognitive changes and that improvement can occur even when there is significant decline in cognitive abilities.

Evidence suggests that areas related to executive functioning including prefrontal and parietal circuits may have greater plasticity and provides explanation as to why this region of the brain may be more amenable to change with aerobic exercise. For instance, Colcombe et al. [44] demonstrated that individuals who were more physically fit had more grey matter volume in the prefrontal, parietal, and temporal regions than less fit individuals. In addition, Marks et al. [45] also reported that more fit older adults had greater white matter integrity preserved in the prefrontal regions than less fit individuals. These cross-sectional studies demonstrated that higher levels of aerobic fitness preserve brain volume and white matter integrity in older adults.

There is also evidence of functional changes occurring in the brain following an aerobic exercise intervention. In a study using functional magnetic resonance imaging, Colcombe et al. [46] found that individuals participating in a 6-month aerobic exercise program had increased neural activity in the frontal and parietal regions of the brain that reflect attention control and a decrease in the dorsal region of the anterior cingulated cortex, with no improvement and a slight decline in cortical volume observed among the controls. Other studies have shown consistent findings of similar neurophysiological changes reflective of physical fitness levels. Findings from another study showed that a 3-month aerobic exercise intervention enhanced cerebral blood volume in the dentate gyrus of the hippocampus, reflected in cognitive testing as improved verbal learning and memory scores [47]. Findings suggest that even short exercise programs can begin to restore losses in brain volume associated with normal aging.

Similar to studies in HF, higher physical fitness is associated with improved physiological changes that enhance cognitive functioning in older adults. For example, in a cross-sectional study of older adults, Erickson et al. [48, 49] used magnetic resonance spectroscopy to examine whether higher aerobic fitness levels were associated with greater concentrations of N-acetylaspartate (NAA) in the prefrontal cortex and determine its effects on improving cognitive function. NAA is a neuronal-specific osmolyte localized to the cell body and is associated with facilitating neuronal energy metabolism, myelination, and regulating neuronal volume. NAA, therefore, is useful as a marker of neuronal viability and neurogenesis. They found that in older adults NAA levels in the frontal cortex correlated with improved working memory performance and that NAA significantly mediates the associative relationship between cardiorespiratory fitness and improved working memory [48, 49]. Therefore, the aerobic-exercise induced improvements in working memory appear to be mediated by neurogenesis and neuronal viability.

The optimal dose of exercise responsible for changes in cognitive functioning has not been established. However, Larson et al. [50] assessed the frequency of participation in a variety of physical activities (e.g., walking, bicycling, and swimming) over approximately 6 years in 1740 older adults. After adjusting for age, sex, and medical comorbidities, older adults who exercised more than three times per week during initial assessment were found to be 34% less likely to be diagnosed with dementia than those who exercised fewer than three times per week. Similar findings between exercise and dementia have been reported in other studies [51, 52]. The intensity level of exercise that provides the most benefit has also not been clearly established. However, Ruscheweyh et al. [53] showed no differences between participants who walked at moderate intensity versus those who were involved in a low intensity gymnastic program on memory function. Both exercise groups had superior memory function scores compared to the sedentary controls. In addition, changes in self-reported activity level correlated positively with improvements in episodic memory as well as with changes in the gray matter volume of the prefrontal and cingulated gyrus. These findings indicate that even low intensity exercise may have beneficial effects on cognitive functioning and neuroplasticity.

Lower risk for cognitive decline is also reported for physically active older adults without diagnosed dementia. In a study examining 349 older adults over 6 years, both subjective and objective measures of cardiorespiratory fitness were collected [54]. Individuals who demonstrated higher levels of fitness at the initial assessment showed greater benefits on standardized cognitive tests at the final assessment, and the relationship between fitness and cognitive function was more robust for the objective measure of cardiorespiratory fitness than subjective measurement. There is also evidence that beginning exercise in middle age may reduce the risk for cognitive decline in later life. Richards et al. [55] examined a cohort of 1919 middle aged adults over a period of 17 years. Participants who engaged in regular exercise at age of 36 and 43 had the lowest rate of memory decline at age 53. These findings indicate that continuous exercise is necessary to optimize cognition across the lifespan. Imaging studies have also complemented these findings by showing a relationship between physical activity at middle age and gray matter volume in later life. Rovio et al. [56] demonstrated gray matter volume to be higher among more physically active individuals using MRI imaging 21 years later. Gray matter volume was the highest among those reporting exercise at least twice per week at midlife compared to those who exercised less frequently. These findings suggest that being more physically active in middle age may have protective effects on cognitive function in old age.

Whether exercise improves memory has been the focus of recent studies. Aerobic exercise appears to reduce hippocampal volume loss. Erickson et al. [48, 49] showed that following a one-year walking program, participants had an increase in hippocampal volume, while those in the stretching control group experienced a decline in gray matter. In another study by Ruscheweyh et al. [53], however, there was no significant difference in verbal memory in adults of 50 years of age or older who exercised versus controls. Although there are few exercise studies among those affected by Alzheimer's disease, evidence suggests that they may also benefit from exercise. Lautenschlager et al. [57] conducted a randomized trial of 170 older adults with either MCI or self-reported memory complaints over a 24-week period. Participants in the exercise group had better delayed recall of word lists and fewer negative cognitive symptoms compared to controls at the conclusion of the intervention.

In summary, although most evidence supports aerobic exercise benefits cognitive functioning, there are some studies that have shown equivocal results. Several potential reasons for these conflicting findings include differences in prescribed exercise, how cardiorespiratory fitness and cognitive functioning were measured, and the age, health status, gender, and fitness levels of participants. Several meta-analyses in recent years, however, have shown a consistent and positive relationship between aerobic exercise and cognition. Importantly, findings from meta-analyses have shown a moderate effect size (>0.5) from aerobic training, which is similar for both normal and cognitively impaired adults. The benefits of exercise training seem to be greater for executive functioning than other cognitive processes. This observation is promising given that executive functioning shows declines with aging but appears to be amenable to improvement with aerobic exercise.

## 6. Animal Models: Evidence for Molecular Pathways

Animal studies have provided the most robust evidence that aerobic exercise improves cognitive function via synaptic plasticity, angiogenesis, release of neurotrophins, and neurogenesis. Synaptic plasticity describes the brain's capacity to modify its neural circuits in response to changes in environmental stimuli in a region-specific manner by enhancing or depressing synaptic transmission over a short or long time period and plays a key role in the development of new neural circuits [58]. Improvements in learning on water maze tasks have been associated with an increased production of neurotrophic molecules, such as brain-derived neurotrophic factor (BDNF), as well as other molecular changes. A number of factors link BDNF to improved cognitive function in exercise including its involvement in neuroprotection and the promotion of cell survival, neurite outgrowth, and synaptic plasticity [59]. For example, when BDNF is administered, there is increased cell proliferation in the hippocampus, while blocking BDNF reduces cell proliferation. Animal models have shown that exercise increases mRNA and protein levels of BDNF in the hippocampus, cerebellum, and frontal cortex, and blocking BDNF to its receptor site eliminates any beneficial effects of exercise induced performance on the Morris water maze [60]. These findings provide clear evidence that exercise increases BDNF levels and appear to be associated with behavioral improvements observed with an exercise program.

In addition to BDNF, other molecular changes in the brain occur as a result of exercise. Insulin-like growth factor-1 (IGF-1), for example, is essential for exercise induced angiogenesis [61] and neurogenesis [62]. In addition, IGF-1 helps to moderate vascular endothelial growth factor (VEGF). When IGF-1 is blocked, there is a significant reduction of new capillaries formed. Furthermore, blocking the influx of VEGF into the brain eliminates exercise-induced neurogenesis, but baseline levels remain unchanged [63]. One function of new capillaries is to deliver necessary nutrients to existing or newly dividing neurons. In relation to this, exercise increases both cell proliferation and cell survival.

Evidence indicates that some neurotransmitter systems are influenced by exercise. For example, serotonin [64] and acetylcholine [65] levels are increased in the brains of exercising rodents, and medial septal GABAergic neurons are suggested to play a key role in the influence of exercise on cognitive processes [66]. BDNF has been shown to regulate dopaminergic and cholinergic neurotransmitters and likely has an important role in the exercise induced changes observed on selected neurotransmitters [67].

Many molecules and molecular pathways are enhanced with exercise that bring about positive changes in learning and memory processes, cortical structure, angiogenesis, and cell proliferation in rodents. It is unclear whether the same molecular mechanisms are present in humans and animal models. It will be important in future human studies to evaluate biomarkers in both serum and brain to determine whether similar changes seen in animal models are occurring in humans as a consequence of exercise participation. In addition, genetic variation in genes, such as BDNF, may be an important factor in determining exercise effectiveness on cognitive outcome variables. Characterizing the genetic profiles of those who benefit the most or least from an exercise intervention is also an important area of future investigation. It is also unclear whether the e4 allele on the APOE gene, a gene associated with the development of cognitive decline and Alzheimer's disease, influences the effect of exercise on cognitive performance [68].

## 7. Implications and Future Directions

Exercise has been shown to improve the deleterious peripheral changes that contribute to disease progression and worsening symptom severity in persons with HF. The underlying mechanisms for improvement in cognition, however, remain poorly understood but likely related to improved cardiac function, cerebral perfusion, and oxygenation, although this link has not been clearly established. Cognitive performance should be evaluated routinely in persons with HF, particularly when first diagnosed and when changes in treatment regimen occur, and with worsening disease severity since these events have been shown to precede changes in cognition [5, 6]. Currently, there are no clinical guidelines to provide recommendations when cognitive screening should be initiated in HF or the best measure and threshold to use for this purpose [69]. If a person with HF screens positive for CI, there should be additional neuropsychological testing conducted to determine type and level of impairment as well as resources available to optimally support the person to be functionally independent of as long as feasible. Earlier identification of impaired cognition may enable clinicians to reduce the risk associated with poor self-care such as simplification of medication regimens or providing resources to individuals to help manage other required lifestyle changes in order to optimally manage their HF.

Well-designed trials are needed in the future with attention to measurement of cognitive function. A broad range of measures have been used to evaluate cognitive function in persons with HF, many of which were designed to be screening measures, which makes interpretation and type of CI difficult. Few studies in HF have included standardized neuropsychological batteries to assess cognitive function. Among those that have, only one [70] included an alternative form of testing suggesting that many studies may have introduced bias via a learning effect. The use of control groups or comparison groups is lacking in many studies, making assessment of cognitive change problematic since many reported positive change in cognitive assessment. Among those that used comparison groups, such as other types of CVD, no significant differences were noted indicating that change in cognition may be a reflection of general worsening cardiac function and may not be specific to HF [5, 6]. In addition, the optimal amount or dose of aerobic exercise, after enhance cognition, has not been clearly established. Preliminary evidence suggests, for example, that aerobic and resistance exercise may affect different neurocognitive pathways and a combined approach may be superior to either approach used alone for improving cognition (see [40, 42, 71]). Few trials have extended beyond 6 months, so whether exercise retains its beneficial effect or has a more robust effect on cognition over longer periods of time is unknown [39].

Screening that measures executive function may provide the most practical method for clinical settings since declines in this domain are well established to contribute to poor outcomes in persons with HF. The Montreal Cognitive Assessment (MoCA) evaluates multiple domains including attention, memory, language, and executive function [72], all of which are impaired in HF. The MoCA lends itself to use in clinical setting because it is brief, requires little training to administer, and is easy to interpret. This instrument has been used to assess MCI in persons with HF successfully and had higher reported sensitivity than the MMSE. Cameron et al. [73] reported that a greater number had lower MoCA scores compared to MMSE scores (66 versus 30, *P* = 0.02). In addition, 41% of patients who were determined to be cognitively impaired by the MoCA were not captured by the MMSE. The MMSE has been criticized as an inadequate screening test for persons with vascular cognitive impairment because of its insensitivity to visuospatial and executive function deficits. The MoCA was designed to be more sensitive to such deficits and thus may be a more sensitive screening instrument for persons with HFFHF. These findings support the use of the MoCA in persons with HF, but more study is warranted in larger and more diverse HF samples.

In conclusion, exercise appears to exert a positive effect on cognition, in particular executive functioning, and may also have a protective effect against cognitive decline with aging. Physiologically, exercise induced BDNF and IGF-1 have been shown to enhance neurogenesis and plays a central role in improving cognitive performance. Further research is needed to better understand the underlying molecular mechanisms of the influence of exercise on cognitive function. Substantial neuronal loss has occurred by the time dementia is diagnosed and currently there are no therapies to replace or generate neurons. There is a great need, therefore, to develop strategies to slow or reverse early onset changes in cognitive decline before significant, irreversible losses occur. Exercise is an inexpensive, practical intervention that may prove to be an effective strategy for slowing or reversing cognitive decline in older persons with HF and in turn may lower the burden associated with these highly prevalent and expanding comorbid conditions.
